# Older men's perceptions of the need for and access to male-focused community programmes such as Men's Sheds

**DOI:** 10.1017/S0144686X16001331

**Published:** 2016-12-21

**Authors:** MARY ANNE NURMI, COREY S. MACKENZIE, KERSTIN ROGER, KRISTIN REYNOLDS, JAMES URQUHART

**Affiliations:** * Department of Psychology, University of Manitoba, Winnipeg, Canada.; † Department of Community Health Sciences University of Manitoba, Winnipeg, Canada.; ‡ Department of Social Work, University of Manitoba, Winnipeg, Canada.

**Keywords:** ageing, community health, mental health, men, masculinity

## Abstract

Although participating in community social programming is associated with positive physical and mental health outcomes for older adults, older men participate less often than women. Men's Sheds is a community programme used primarily by older men that originated in Australia and is well established there. The goal of the current study was to explore men's perceptions of the need for Men's Sheds and issues concerning access to them in Canada, a country with a small but growing Men's Sheds movement. We conducted focus groups with 64 men aged 55 years and older, including Men's Sheds members and men from the community who were unfamiliar with this programme, and analysed the data using the framework analytic approach. The data revealed two primary themes concerning: (a) the need for male-focused community programmes, including the sub-themes reducing isolation, forming friendships and engaging in continued learning; and (b) access to programmes, including the sub-themes points of contact, sustaining attendance and barriers. Findings suggest that in order to reduce the likelihood of isolation and increase opportunities for social engagement, exposure to the concept of male-focused programming should begin before retirement age. In addition, such programmes should be mindful of how they are branded and marketed in order to create spaces that are welcoming to new and diverse members.

## Introduction

Participation in community activities is associated with positive physical and mental health outcomes for older adults, including increased lifespan and resiliency in the face of age-related changes, such as loss or declining health status (Agahi, Silverstein and Parker 2011; Milligan *et al.* 2013). Older adults who participate in community programmes may experience these positive health outcomes as a result of increased social connection and support (Agahi, Silverstein and Parker 2011; Milligan *et al.* 2013). Although social support needs can be met in a variety of ways, access to community-based programmes is one important avenue that supports building social connections for some older individuals (Cohen-Mansfield and Frank 2008; Dickens *et al.* 2011). Despite the benefits of engaging in community programmes, older men do not often participate or are reluctant to join (Milligan *et al.* 2013). Similarly, older men are especially unlikely to seek professional help when they are struggling with mental health concerns (Mackenzie, Gekoski and Knox 2006; Corna, Cairney and Streiner 2010; De Leo *et al.* 2001). Our study contributes to a body of research aimed at better understanding the need for male-focused community programmes, and possible barriers to participation.

The extant research has highlighted gender differences in community programme participation. For example, the systematic review of Milligan *et al.* (2013) related to the impact of community programming on men's health and wellbeing indicated that older men participate less often than women and that much of the existing programming tends to be more appealing to women. Interviews with older men in the United Kingdom (UK) revealed that they participated in organised activities less often overall, and that those who did tended to be younger, married and from middle-class backgrounds (Davidson, Daly and Arber 2003). This may have important implications for men as they age, since reluctance to participate in community programmes may contribute to social isolation (Milligan *et al.* 2013). Access to transportation, language barriers, disability and chronic illness are other barriers that can prevent some older adults from participation in social activities (Marx *et al.* 2010; Stewart *et al.* 2011). These barriers, combined with gender socialisation that leads many men to value masculine ideals such as autonomy, strength and independence (Mansfield, Addis and Mahalik 2003; Real 1997), may contribute to older men's lower rates of help-seeking and programme participation.

In light of the benefits of engaging in community programmes and men's relatively lower rate of involvement, researchers and advocates of men's health and wellbeing suggest there are benefits to developing community programmes that target older men in particular. One such programme is Men's Sheds – these are grass-roots, community-based groups that began in Australia in the 1990s and have since expanded to other countries, including New Zealand, the UK, Sweden, Denmark and Canada (Golding 2015). Men's Sheds provide men with opportunities to socialise while participating in ongoing learning and activities such as woodworking, repair projects and community volunteering (Golding 2015; Martin, Wicks and Malpage 2008; Morgan 2010; Ormsby, Stanley and Jaworski 2010). A growing body of research suggests that Men's Sheds have a positive impact on social engagement, adult education and health (Ballinger, Talbot and Verrinder 2009; Beyond Blue 2013; Carragher 2013; Cordier and Wilson 2014*b*; Culph *et al.* 2015; Golding 2015; Moylan *et al.* 2013; Reynolds *et al.* 2015; Wilson and Cordier 2013). However, Men's Sheds have predominantly been attended by working-class, Christian men in Australia, Ireland, New Zealand and the UK (Golding 2015; Milligan *et al.* 2013). This raises important questions about the need for such programmes and barriers to involvement in other countries, cultures and diverse sub-sets of older men.

There is a small but growing body of research suggesting that there is interest in Men's Sheds within diverse groups and in countries that do not currently have strong shed cultures. In Australia, sheds have been shown to reduce social and health inequalities among some marginalised groups (Beyond Blue 2013; Misan, Haren and Ledo 2008). One study revealed that reduced pressure to adhere to work-related identities and an inclusive environment were seen as beneficial by Men's Sheds members living with disabilities (Hansji, Wilson and Cordier 2015). Men's Sheds have also been identified as an effective way to promote health and to reduce barriers to health and social services for Indigenous men who preferred to access health information at men's groups rather than traditional health-care settings (Southcombe, Cavanagh and Bartram 2014). It is also important to examine factors affecting involvement in sheds outside Australia, Ireland, the UK and New Zealand, where sheds have the longest history and greatest availability and recognition (Golding 2015). A recent study from central Canada explored characteristics and experiences of Men's Sheds members that affected their initial and continued involvement in sheds (Reynolds *et al.* 2015). This study found that men with certain values (*e.g.* pro-social attitudes and the need to keep busy and strive for achievement), who were isolated and lonely, and whose friends and family encouraged them to join were most likely to get involved in sheds. Participants identified family responsibilities, physical limitations and transportation difficulties as potential barriers to involvement. With this study's primary focus on men who were already involved in a Men's Shed, the perspectives of men who are not currently involved in Men's Sheds or other male-focused community programmes are therefore important to uncover in order to better understand whether male-focused programmes appeal to a broader and more diverse range of older men.

The aims of the current study were to describe older men's perceptions of the need for male-focused community programmes in the example of Men's Sheds and potential issues in accessing them. These perceptions come from older men who were involved in Canada's first Men's Shed, as well as from men who had never heard of Men's Sheds, including men from a variety of socio-demographic backgrounds. This study is, therefore, particularly relevant to international contexts where the Men's Sheds movement is less well developed.

## Methods

### Study design

We employed qualitative focus group methods for this study. Focus groups allowed us to explore a range of perspectives while also allowing for an exploration of group interactions and culture (Liamputtong 2011). Most participants attended three focus groups (we describe the purpose of these groups below), allowing them to develop rapport with one another and the group facilitators. While challenges exist related to focus group data collection (*e.g.* some group members may speak and share more than others), this method was considered appropriate because it allowed us to hear from a relatively large number of shed and non-shed participants, and to build on the content of previous sessions during subsequent ones.

### Recruitment

We recruited men aged 55 and older with mixed knowledge and experience with Men's Sheds in Winnipeg, Manitoba, Canada. First, we recruited men with knowledge of Men's Sheds from Men's Shed Manitoba, the first Canadian Men's Shed, with help from the shed's co-founder. The co-founder, who did not take part in the focus groups, personally invited members to take part and included information about the study in the shed's weekly newsletter. We also employed snowball sampling, where men were welcome to invite other members to take part. Second, we recruited men with no knowledge of Men's Sheds using three methods: (a) a one-day advertisement in several local community newspapers, (b) a research assistant visited local coffee shops within diverse neighbourhoods in Winnipeg; and (c) directors of diverse community organisations in Winnipeg and one rural area helped to recruit their members. These community organisations included: (a) Aboriginal Health and Wellness Centre of Winnipeg, Inc. (AHW), which houses a programme for Aboriginal men called Men's Healthy Living; (b) Rainbow Resource Centre (RRC), an organisation that provides services to the lesbian, gay, bisexual and trans (LGBT) community in Winnipeg and surrounding area; (c) Springfield Rural Seniors Centre (SRS), which provides services to seniors living in the rural area of Springfield; (d) Welcome Place Settlement Agency (WP), an organisation that provides resources to recent immigrants to Canada; and (e) Man Coffee, a local Winnipeg coffee group for men.

### Sample

Sixty-four men took part in the study, including 22 Men from Men's Sheds Manitoba, two men from local coffee shops, 20 men who responded to the one-day newspaper advertisement and 20 men from five community organisations. For an overview of socio-demographic characteristics, *see* Table 1.

**Table 1.** Socio-demographic characteristics

	Total N (%)
Age:	
Under 55	7 (12)
55–59	7 (12)
60–64	12 (21)
65–69	15 (26)
70–74	13 (22)
75–79	1 (2)
80–84	3 (5)
Education:	
Less than high school	5 (9)
High school	16 (27)
Technical or college	15 (26)
University/bachelors	12 (21)
Post-graduate, Masters	10 (17)
Current occupational status:	
Full-time	3 (5)
Part-time	5 (9)
Retired	41 (71)
Disability leave	2 (3)
Unemployed	4 (7)
No response	3 (5)
How long have you been retired?:	
0–1 years	6 (10)
1–2 years	7 (12)
2–5 years	7 (12)
5+ years	21 (36)
Not retired	10 (17)
No answer	7 (12)
Current household income (Can $):	
0–19,999	7 (12)
20,000–34,999	11 (19)
35,000–59,999	21 (36)
60,000+	15 (26)
No response	4 (7)
Marital status:	
Single	11 (19)
Common law	4 (7)
Married	31 (53)
Widowed	5 (9)
Separated	3 (5)
Divorced	4 (7)
Racial/ethnic group:	
White	53 (91)
Aboriginal, First Nations, Inuit	2 (3)
No response	3 (5)
First language:	
English	52 (90)
Polish	4 (7)
French	2 (3)
Self-rated mental health:	
Poor	3 (5)
Fair	8 (14)
Good	24 (41)
Very good	19 (33)
Excellent	4 (7)
Self-rated physical health:	
Poor	3 (5)
Fair	8 (14)
Good	24 (41)
Very good	19 (33)
Excellent	4 (7)
Have you ever experienced a significant problem with stress, anxiety or depression that interfered with your ability to function on a daily basis?:	
Yes	27 (47)
No	31 (53)
Have you ever sought professional help for a problem with stress, anxiety or depression?:	
Yes	30 (52)
No	28 (48)
Are you currently involved in any community programmes or activities?^1^:	
Yes	24 (57)
No	12 (29)
No response	6 (14)

*Note*: 1. This question was only asked of participants not involved in Men's Sheds or community organisations.

### Study procedures

We obtained ethics approval through the University of Manitoba Research Ethics Board. Data collection took place from March to October 2014. All participants provided informed consent and completed a socio-demographic survey that also included questions about mental health status and participation in community programmes. A research assistant took field notes at each focus group that captured group dynamics, when/how mental health was talked about and topics for consideration in subsequent focus groups. Focus groups were audio-recorded and transcribed.

In Phase One of the project, two groups of Men's Sheds members (N = 12 and N = 8) and two groups of participants who had no knowledge of Men's Sheds that we recruited through coffee shops and newspaper advertisements (both N = 9) completed initial focus groups. Men's Sheds participants discussed how they heard about Men's Sheds and how the group developed; participants without knowledge of Men's Sheds discussed their knowledge of community programmes more broadly, and whether there is a need for male-focused community programmes specifically. Participants in all of the Phase One focus groups also discussed how organisations such as Men's Sheds could be developed, marketed and made most accessible to older men.

In Phase Two, several months later, these same four groups met for a second focus group in which they reviewed and verified a summary of results from Phase One that our team provided. Men's Sheds participants in this second phase discussed activities that their members are interested in, how their group is branded and marketed, and how they reach out to potential new members. Non-Men's Sheds participants provided feedback on their first impressions of Men's Sheds’ name and imagery related to visual branding, and they discussed what information should be included in materials geared towards starting groups for older men, and where these would be made most accessible. They were also asked about the types of activities they would like to do and whether others in their lives would support the idea of men's programmes.

In Phase Three, we reviewed field notes and audio files from the first two phases and prepared a detailed summary of the results. Approximately four months after the second round of focus groups, participants completed a third focus group in which they commented on the summary of results and also broke into small working groups and provided written comments on paper copies of the summary that we provided.

In Phase Four, we conducted new focus groups with men from five diverse community organisations that we described in the Recruitment section. This additional recruiting took place as a result of a lack of apparent diversity among the focus group participants in Phases One to Three. These focus group participants discussed their impressions of the need for male-focused programming, Men's Sheds’ name and imagery, what information should be included in materials geared towards starting groups for older men and where these would be made most accessible.

### Data analysis

We used the framework analytic approach (Ritchie *et al.* 2014) to analyse the focus group data. This approach is commonly used in applied policy research, and emphasises presenting the participants’ responses without theoretical interpretation of the findings (Srivastava and Thomson 2009). Since the data collected in this study were used to develop a toolkit for starting new sheds, framework analysis was considered appropriate. Framework analysis involves six steps: (a) familiarisation, (b) identification of a thematic framework, (c) indexing and sorting, (d) reviewing data extracts, (e) data summary and display, and (f) abstraction and interpretation. It should be noted that all steps are not necessary, depending on the research question (Ritchie *et al.* 2014). We familiarised ourselves with the data by having two team members review the field notes and audio files as they became available after each focus group. In the second step, these team members read focus group transcripts and developed the initial framework. In the third indexing step, two members of the research team used NVivo 10 to organise quotes from the transcripts according to their respective themes. In the fourth step, the themes and sub-themes were then further refined by other members of the team during weekly meetings to ensure that the extracted quotes were grouped in the way that best reflected the transcripts. Finally, in the fifth step, the research team met to review and finalise the themes and sub-themes that are presented in the Findings section.

### Quality of research process

At every stage of the process, our inter-disciplinary team discussed the data and the emerging themes, which is considered peer review (Braun and Clarke 2013). This peer-review process was considered important because older men's perceptions of needs and access to male-focused community programmes has not been well explored in the Canadian context. Therefore, hearing multiple perspectives by research team members allowed for a more nuanced understanding of the findings before naming and describing them in the final themes and sub-themes. We also collected multiple forms of data (field notes, transcribed audio-recordings and written participant data), which allowed the research team to triangulate, ensuring that common themes across data-sets were tended to, or reviewed, when there appeared to be anomalies. We kept the main research question and extant literature review in mind throughout the research process, adding to theoretical sensitivity on the topic. The focus groups themselves were designed as a ‘member checking’ feature – so that we received detailed and ongoing feedback from our participants about their views of the data and emergent themes during second and third group sessions.

## Findings

**Figure 1.**
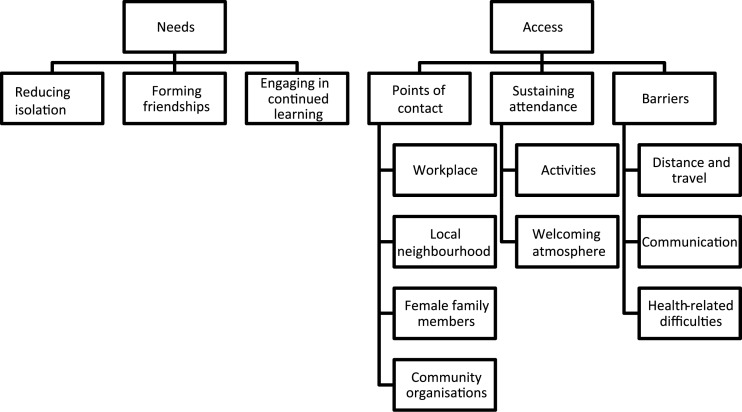
Summary of findings.

Findings have been organised into two themes and accompanying sub-themes that describe, firstly, participants’ perceptions of the needs for and, secondly, their access to male-focused community programming. Throughout this section we identify whether quotations come from Men's Shed participants, non-Men's Shed participants, or from participants from one of the five community groups by using the community groups’ acronyms from the Methods section. For an overview of themes and sub-themes, *see* Figure 1.

### Needs

A key topic of discussion within our focus groups was the need for male-focused community programming, primarily in relation to the example of Men's Sheds. This theme ‘needs’ includes the sub-themes *reducing isolation, forming friendships* and *engaging in continued learning*.

#### Reducing isolation

The experience of isolation emerged as an important factor contributing to the need for community programming across all focus groups. Participants discussed personal experiences with social isolation as well as the experiences of close others who have few opportunities for social contact, noting that this would be a primary reason to develop programming for men. One AHW participant summarised this theme when he explained, ‘There's a lot of us guys just sitting at home doing nothing. They're looking at the four walls and they don't have anybody to talk to.’ While participants across all focus groups identified a need for community programming to reduce isolation, it is noteworthy that there was some ambivalence around the idea of groups for men specifically. For example, one participant stated, ‘I find it difficult separating the two and saying that you guys are strictly a man's group’ (non-Men's Shed participant). However, participants also expressed that men tend to be less likely than women to form friendships and social connections actively. For this reason, men viewed the focus on groups for men as positive and beneficial.

Participants talked about life situations that contributed to men's isolation. For example, men talked at length about retirement and the loss of an established group of colleagues. In this situation, the idea that isolation may be particularly relevant for men, compared to women, emerged. Many participants explained that men tend to strongly identify with their employment, leading them to focus less on establishing relationships outside this context:

I don't mean to stereotype men, they're very career orientated, and when that gets taken away there's a big void … is there any transition programmes where you connect and you don't lose that connection? (non-Men's Shed Participant)

Other participants discussed the dearth of places men can go to for social contact. One participant from AHW said, ‘for me it would be really beneficial because of where I live. I see so many people, even my age, they spent all day sitting in their room.’ Some participants who were from Welcome Place and those who were new to Canada explained that the concept of Men's Sheds could invoke feelings of family and support.

Men's Shed participants talked about past experiences of being at home alone and working on projects independently. This sense of aloneness was a motivator for them to join the Men's Shed. These men felt that Men's Sheds could benefit those men who are the most isolated. One Men's Shed participant said:

I see the need, it is just immense and the guys who should be here aren't here. There's so many people sitting around having a coffee and they don't know anything else. But the need is big and I enjoy taking part.

#### Forming friendships

The second sub-theme that emerged was the potential for men's groups to be a place where men can form friendships. Participants emphasised the importance of having people to talk to and develop friendships with, however, they also explained that men are less likely than women to develop friendships outside those that are facilitated by their female partners. They voiced that men in long-term relationships with women may not be actively involved in maintaining social networks throughout their lives, but that groups for men could help to create opportunities for them to do so. One participant explained that, while men can participate in a variety of activities with their female partners, there would be a benefit to participating in these activities in the context of a male-focused group because:

To make it an exclusive male group so that you don't get into the situation where you become divorced or your wife passes away and have to put that work into it, and I think a group focused on men is self-perpetuating because then the people participating get more practice in being responsible for their own social network, their own social life, instead of that doing, that thing through the female spouse. (non-Men's Shed Participant)

#### Engaging in continued learning

Finally, participants expressed that their need to engage in continued learning could be met through male-focused community programming. Activities involving learning and teaching were listed as highly appealing across all focus groups. The idea of sharing knowledge or skills acquired through work or knowledge related to participants’ cultural backgrounds were particularly appealing. One Men's Shed participant said, ‘I mean somebody else knows how to do glass, somebody else knows how to fix motorcycles, so I kind of like that idea if there's an opportunity to learn something new.’ Similarly, other participants discussed their desire to learn new skills from one another and through mentorship. One participant discussed his experience ‘learning how to pitch a tee pee, or a tent – add a little bit of that knowledge in there’ (AHW). Participants explained that teaching and learning from each other would be a primary reason to attend Men's Sheds or other men's groups. One participant explained to the rest of the focus group members:

How would you like to learn about estate planning, well I could tell you a lot about that and it may be helpful to you, I don't mind sharing that information. I feel good being able to explain to somebody something they may not know, similarly you could teach me the art of throwing horseshoes. (non-Men's Shed participant)

There was also interest in learning about topics through listening to guest speakers. One participant spoke about guest speakers as a potential activity in community programming, stating, ‘a guest speaker to provide insight into a particular aspect of life would be of interest to me’ (non-Men's Shed participant). For members of Men's Shed Manitoba, this was already a part of their regular programming.

### Access

The second major theme that emerged through the focus groups was the issue of having ‘access’ to male-focused community programmes. This theme included three sub-themes: *points of contact, sustaining attendance* and *barriers*.

#### Points of contact

This first theme focused on ways of letting men know about male-focused programmes such as Men's Sheds. Many participants highlighted the potential benefits of learning about and receiving information on male-focused programming in the workplace when they are close to retirement. Men noted that this could act as a potential preventative measure for the development of loneliness and social isolation, which can occur following retirement and loss of established social networks. The workplace could act as a central point of contact for men, to let them know about options for community programmes that they might enjoy. This was expressed as equally important to financial planning for retirement as it was explained:

As somebody would approach retirement … that would be the ideal time as you say whether it's on a brochure or printed sheet or these are available for you, here's a bridge club, here's this, you know just different things like completely different things that maybe that individual would key in on one and he says, oh, okay well they meet every Tuesday, here's a name the president of whatever and the phone number and take it from there. That's in, in my mind almost as important as the guy telling me that uh you know you've got uh $5,000.00 dollars a month for, for life you know, uh because that you know is coming in, but okay now what am I gonna do, you know. (non-Men's Shed participant)

Local neighbourhood contacts were discussed as another point of contact for older men to learn or hear about male-focused community programming. Participants indicated that they would most likely learn or hear about community programmes through a combination of local sources such as community newspapers, community mailboxes, local signs and word of mouth. Most participants felt that, while the Internet is important, it is not currently the first point of contact for older men. Participants also noted that information on the internet is not readily accessible to all men, given varying comfort levels and access to computers. One non-Men's Shed participant explained, ‘I access the computer, I like some things in print. I think that you have to just keep it simple.’ Emphasis on a combination of local contacts was further exemplified when participants described that announcements in newspapers also have limited reach:

There's the newspaper, but then you have to subscribe. There's websites but you have to know how to access the internet – really sophisticated. But maybe that's another thing that keeps us isolated as we get older is we are forced into a communication age that we don't understand. (non-Men's Shed participant)

Similarly, the importance of a direct invitation from a neighbourhood Men's Sheds advocate or leader was a common point of contact for some Men's Sheds participants. These participants described how the friendly group leader was a driving factor in their initial attendance. For example, one participant said, ‘Without talking to him I probably wouldn't have come. He knows how to talk you into things. I had no expectation when I came here, but I stayed.’

Female family members were also perceived as an important point of contact. Some non-Men's Sheds and Men's Sheds participants indicated that they were invited to join groups or activities by their daughters or wives. One participant explained that his attendance at the focus group for the current study was the result of his wife's prompting, saying ‘I saw the ad and I probably would have looked at it and gone on to something else, except my wife said “this is for you, look at it”’ (non-Men's Shed participant).

Community organisations, including health-care service providers, were also perceived as important points of contact. Participants might find written information, such as notices or posters, at community organisations or hear about programmes from health-care providers. For example, a Men's sheds participant had heard about the Men's Sheds programme through a visit to his therapist, explaining:

Well I was going through some counselling to figure out what I want to do and having a rough time at work. A counsellor offered maybe this may be something I'd be interested in, so I just took a look at it. (Men's Sheds participant)

Similarly, an AHW participant said that his primary means of finding out about community events would be at the centre through which he participated in the focus group.

#### Sustaining attendance

Participants described that both the activities and a welcoming and friendly atmosphere would keep them coming back after initial programme attendance. Participants explained that there would have to be a common interest in the activity or activities taking place in order to keep them coming back:

It's not about only men or only 55 plus. It's that the group brings in people with some sort of common interest, whether it's to learn something they already know or can teach. That was the thing that caught me. (non-Men's Shed participant)

Finally, a welcoming and friendly atmosphere of a male-focused community programme was described as a key element to initiating and sustaining attendance. In focus group discussions with participants, the idea of the ‘buddy system’ emerged, whereby a current group member would be in charge of introducing first-time members to everyone and orienting them to the group so that new members’ first experience was positive and they would want to keep coming back. One participant said:

Welcoming is the most important thing when they arrive at the group. If you don't feel welcome you're not going to go back to it. If somebody comes up and greets you, it tends to be more encouraging. (non-Men's Shed participant)

The significance of a welcoming atmosphere in choosing to join or remain with a group or programme was highlighted by some participants’ past decisions to leave programmes when they felt unwelcome. For example, men who did not identify as heterosexual expressed a concern about social exclusion because they have historically been excluded from ‘men's’ spaces. One RRC participant said ‘even though we had something in common with the straight group we always had that in the back of our minds – am I going to be chastised or welcomed? Fortunately, the 99 per cent was welcoming and there's always one in either group that is not’ (RRC participant).

A friendly atmosphere was also underscored, as some participants related men's lack of friendliness with feeling unwelcome. One participant said ‘as long as the group is recent it's good, but men are not welcoming, so if you take a club that's been going four, five years and you come in alone, forget it’ (non-Men's Shed participant). Similarly, a SRS participant said, ‘I think women bond quick. If you've got a social occasion the women chat much easier, and the men stand back.’ While Men's Sheds participants discussed that feeling unwelcome had been a barrier for them in prior group involvement, they perceived Men's Sheds to be welcoming to all individuals, including women. One Men's Shed participant explained that groups other than Men's Sheds were ‘cliquey, and it's quite the opposite here. It's open and welcoming’ (Men's Sheds participant).

#### Barriers

This theme describes the circumstances and characteristics that would reduce access to male-focused community programming. These included distance and travel, communication and health-related difficulties.

Distance and travel were the most common barriers described by the participants as existing or potential barriers to engagement. Participants considered travelling outside their own neighbourhood as a barrier to participating in community programming. One participant explained that, especially after retiring, he would not travel to a neighbourhood too far outside his own, stating that ‘We're talking about accessibility. If you go to work far away, well okay I'll go. But now I'm retired I'm not going to go very far for a social group’ (non-Men's Shed participant). When Men's Sheds participants were asked to describe how they accessed potential new members to join the group, they noted that most of their members also live close by and heard about Men's Sheds through word of mouth. In discussing the ways in which Men's Sheds might expand, one participant said, ‘I would gladly go to another community to help start a shed, but I don't have the resources to go there every week if it's a 50-mile drive.’

The second barrier to participating in community programmes was communication – specifically the predominant language used to communicate within and about the programme and the materials and messaging chosen to represent male-focused groups. For participants who were newcomers to Canada and still learning to speak English, language was their primary barrier to participation. However, the participants from WP also discussed that groups for men, such as Men's Sheds, could provide opportunities for them to work on their English-language skills.

Focus group discussions on how to promote Men's Sheds revealed that, for some men, the materials and messaging that represent male-focused groups can be barriers to participation. Branding and promotional materials were described as potential barriers because some participants indicated that if they did not like the way male-focused programming was presented they would not seek out more information about it, nor would they attend. With reference to the name ‘Men's Sheds’, one participant said that it reminded him of ‘a little eight by ten out in your back yard for storage. Does that mean something different in Australia?’ (non-Men's Shed participant). Another man had a strong reaction to the idea of going to a place called a shed because he associated the word with discipline, noting ‘with older men, when you say the word shed you think – if I misbehaved Dad put me out in the shed to straighten me out’ (non-Men's Shed participant). Some participants felt that the name was stereotyping. One participant said:

I was thinking of *Red Green* [a Canadian television comedy show about a highly stereotyped man]. It sounds a little stereotypical, like it's a shed word that sounds stereotypical. It's like the woman is in the house looking after the house all day and the children and the guys got the basement and the garage and the shed. (non-Men's Shed participant)

During one focus group there was discussion about logos and images of various Men's Sheds and shed organisations. Many of these logos and images had pictures of tools on them, making some men wonder if they needed to be proficient at carpentry in order to join. For example:

Hammer is implying you're a handy type person. I'm mechanically declined you know, give me a hammer and I'll probably whack myself in the thumb or something, why again does it have to be a physical thing? (non-Men's Shed participant)

Some participants perceived logos that conveyed friendship and community to be preferable to the logos and images that focused on tools and carpentry.

Finally, physical and mental health-related difficulties were talked about as barriers to participation. With regard to physical health, for example, one participant said, ‘I do have a couple of friends whose health has failed and you can really see how they can't do these activities’ (non-Men's Shed participant). Depression was listed as a mental health problem that would be a barrier to participation. An RRC participant described this barrier during a discussion of the ways that male-focused programming could be made most accessible, stating ‘It's chickens and eggs you see. If you're a bit depressed or you're isolated, you really don't want to join anything.’

## Discussion

The aim of this paper was to describe men's perceptions of the need for male-focused community programmes and potential issues in accessing such services. This study captured the views of men who were already involved in Men's Sheds as well as those who were not involved in male-focused community programmes in Canada – a country where Men's Sheds are relatively new and unknown. The two main themes that emerged from the data, concerning the need for male-focused community programmes and access to them, add to existing research aimed at understanding ways to foster social engagement, learning and health among older men.

With respect to the need for programmes such as Men's Sheds, participants emphasised loneliness and social isolation by voicing the degree to which older, retired men were often alone at home or sitting in coffee shops with little to do. These discussions are backed by research showing that between 10 and 40 per cent of middle-aged and older adults report being isolated or lonely (*e.g.* Dykstra 2009; Newall *et al.* 2009; Pinquart and Sorensen 2001) and that isolation and loneliness among older adults is a concern among policy makers. In Canada, the National Council of Seniors declared social isolation as its priority area in 2013 and 2014. Participants in this study overwhelmingly discussed how few community programmes currently exist that focus on older men, and that programmes such as Men's Sheds are one potential avenue for both reducing isolation and loneliness, and for developing friendships. Again, this notion is supported by research suggesting that one of the clearest benefits to involvement in Men's Sheds is social engagement and inclusion (Cordier and Wilson 2014*a*; Culph *et al.* 2015; Fildes *et al.* 2010; Golding 2015; Milligan *et al.* 2013; Moylan *et al.* 2013; Reynolds *et al.* 2015; Wilson and Cordier 2013). Most participants in the current study felt strongly that men are less likely than women to develop and foster social networks throughout their lives. Older men's isolation was perceived as a continuation of this lack of network-building throughout life that simply ‘catches up with them’ once they retire or lose a female spouse. Also the friendships that men developed as a result of their involvement in the shed filled what was, for many, an unmet need in this regard.

The men in the current study also highlighted the need to both learn new information and skills, and to teach and share with others the skills and information that they were proud of. Adult learning has been a significant focus of early Men's Sheds research (Wilson and Cordier 2013) and this topic is also captured in studies focusing on mentorship within sheds (Brown, Golding and Foley 2008; Bulman and Hayes 2011; Cordier and Wilson 2014*a*). That men in the current study focused on this need, but not on the need to improve health, is interesting in light of recent discussions of the importance of demonstrating the health benefits of shed involvement. Our study suggests that this focus on social determinants of health appears to be more of interest to policy makers than to men who take part in sheds (Golding 2015; Wilson and Cordier 2013).

Apart from the clearly expressed need for Men's Sheds, the other primary theme that emerged in the current study addressed accessing such programmes. The points of access sub-theme touched on ways in which lonely or isolated men could make their way to a shed for the first time, including at the workplace, in their local neighbourhoods, from female family members and through community organisations. While research has pointed to the benefits of Men's Sheds in helping men adjust to retirement (Golding 2011, 2015; Martin, Wicks and Malpage 2008; Ormsby, Stanley and Jaworski 2010; Reynolds *et al.* 2015), findings from the present study indicate a preference for learning about male-focused community programmes prior to retirement. Community organisations and health-care providers may be a particularly important access point for men who are living with illnesses or disabilities, Indigenous men in Canada, those who are new to Canada and those who are part of the LGBT community, since these organisations may be a trusted source of information and a place where men are likely to visit. Using community organisations to put men in touch with Men's Sheds may be more effective in Australia, where Men's Sheds are often couched within community health organisations that are recognised as an effective site through which to reach marginalised communities, particularly ‘older and rural men from very diverse, isolated and often disempowered backgrounds’ (Golding 2015: 44). In Canada, existing sheds have developed largely independently from health organisations.

Men's perceptions of barriers to participation in community programming included transportation difficulties, health problems and challenges related to communication. Illness and mobility issues were also identified as potential barriers to engagement in sheds by Reynolds *et al*. (2015). These issues are common concerns in later life and we suspect there is variability within and among countries in terms of the supports that are available to help overcome them. With respect to communication challenges, one issue that came up was some older men not having access to computers or not being computer literature, especially if information about sheds was only available online. Interestingly, a website called Sheds Online demonstrated benefit, but shut down because of decreasing usage (Beyond Blue 2016).

Interesting discussions emerged in the current study with respect to the impact of culture and diversity on access to Men's Sheds. An important factor affecting whether new members will join sheds and remain engaged was whether men felt welcomed and at home during their first visit. We are not aware of previous research examining men's first impressions of Men's Sheds in a Canadian context. The fear of feeling unwelcome emerged prominently among the study participants, and views ranged from the perception (stereotype) that men themselves are unfriendly, to the fear of being excluded, *e.g.* due to their sexual orientation. Relatedly, the ways in which Men's Sheds are branded and promoted emerged as a concern for many of the non-shed men. Some men felt that carpentry-focused logos and images could be stereotyping and unintentionally pushing away men who do not have skills or interests related to this traditionally masculine activity. For participants from community organisation focus groups, the overtly stereotyped male imagery evoked a sense of exclusion and did not promote the idea of a welcoming, inclusive environment. These findings may point to regional differences within Canada, as well as between Canada and other countries in terms of how men conceptualise a ‘men's space’ and how various conceptualisations are branded and marketed in ways that may include and exclude certain groups of men. Although it is unlikely that a single image or brand will appeal to all men, findings from the current study suggest that male-focused community programmes may have broader appeal in Canada if the associated images focus on friendship and learning.

Discussions of diversity and culture affecting access to programmes such as Men's Sheds raise interesting questions about social inclusion of older men in other parts of the world – non-English-speaking countries in particular. Men's Sheds have primarily emerged in Anglo-Saxon countries, including Australia, New Zealand, Ireland, the UK and Canada. While Milligan *et al.* (2013) discussed the need to include older men from diverse cultural groups, the present findings cannot contribute to our understanding of social inclusion of diverse cultural groups from other non-English-speaking countries. While relatively little is known about loneliness in non-English-speaking countries, a small body of evidence is emerging in China, where drastic changes in the last 30 years in fertility, social attitudes concerning family and collectivism, and uneven economic mobility have resulted in decreases in large inter-generational households and in the proportion of older Chinese adults who live with children (Luo and Waite 2014; Wu *et al.* 2010; Yang and Victor 2008). Likely as a result of factors such as these, a recent nationally representative study of 14,072 older Chinese adults found that 28 per cent reported feeling lonely (Luo and Waite 2014). These data suggest that programmes such as Men's Sheds, although perhaps with appropriate cultural modifications, could be valuable for at least certain older men across countries and cultures.

### Strengths and limitations

The current study has many strengths, including its inclusion of Men's Sheds members and non-members, including individuals from diverse community organisations representing recent immigrants, gay men, First Nations men and men from a rural community. The findings of this study therefore contribute to a broader understanding of men's perceptions of male-focused programming from diverse perspectives within Canada, a country with a developing Men's Shed movement. In addition, older men who were not previously aware of community programming or of Men's Sheds took part, allowing for an exploration of Canadian men's first impressions of the concept of Men's Sheds. Another strength of the current study is that while one focus group was conducted with each of the five community organisations, multiple focus groups with the Men's Sheds and non-Men's Sheds participants took place over three time periods. This allowed most participants to develop rapport with one another and the group facilitators during the focus groups, contributing to a rich and open dialogue about men's programming, while also allowing participants multiple opportunities to share their feedback and validate previous focus group findings. This study must also, however, be considered in light of several limitations. It is a relatively small qualitative study in one region of central Canada, so the results may not generalise to older men in other parts of the country, and outside Canada. A second limitation of this study, given the focus on access to community programmes, is that none of the participants appeared to have significant physical or mobility limitations. In addition to this, we encountered challenges related to the recruitment of older men who are the most isolated, and this will be an important consideration for future research.

### Conclusion

The present study contributes to an increased understanding of older men's perceptions of the need for and access to male-focused community programming. There was consensus among participants of the need for men's increased participation in community programmes and of older men's risk of isolation. Our findings echoed previous research on older men's involvement in Men's Sheds, such as a desire to reduce isolation, a preference for activities involving exchange of knowledge, and the importance of men's programmes as a space to develop friendships. However, this study contributes to further understanding of the access and barriers to programming for men, such as the importance of local connections, accessing men before they retire, the importance of branding and imagery, and addressing some of the barriers that may exist to getting older men involved. This study has practical implications for the development of male-focused programmes. Findings related to points of contact for older men indicate that they should be introduced to male-focused community programmes before they retire and that programmes for men may be more accessible to particularly isolated men and those who are living with physical or mental health conditions when they are connected with health and social service organisations. Male-focused programmes should include formal or informal peer supports to greet new participants and ensure they do not need to attend a first meeting alone. This may help alleviate men's concerns around showing up alone to a new programme and feeling unwelcome. Male-focused programmes will also require ongoing and thoughtful consideration of the ways in which they are appropriate for addressing the needs of diverse men, including those in non-English-speaking countries.
